# Supplementary material for: WordCommentsAnalyzer: A windows software tool for qualitative research
Source: F1000Res. 2018 Sep 4;7:536. Originally published 2018 May 3. [Version 2] doi: 10.12688/f1000research.14819.2 (PMC6137411; doi:10.12688/f1000research.14819.2)
Supplement: Supplementary file 3 [file f1000research-7-17614-s0002.tgz › b2a79496-4ada-47c4-9682-a99ce867836d.zip!/Eight Iranian Health Journals/Yafteh _61.docx]

© Yafteh  . This work is licensed under CC BY 4.0 (https://creativecommons.org/licenses/by/4.0/).

Volume & Issue: دوره 5 - شماره 4

Title: همه‏گیری‌شناسی اختلالات روان پزشکی در استان لرستان (1380)

http://yafte.lums.ac.ir/article-1-991-fa.html

مقدمه: بار اختلالات روان پزشکی در کشورهای پیشرفته تا حد زیادی با پرسش‌نامه‌های غربالگری و مصاحبه‌های بالینی ساختاریافته شناسایی شده است؛ ولی بار اختلالات روان پزشکی در کشور ما محدود و تعداد مطالعات انجام شده در این زمینه اندک است. برنامه‏ریزی برای ارائه خدمات اساسی بهداشت روان به افراد، نیازمند آگاهی از وضعیت موجود بیماری های روانی در جامعه است. این مطالعه با هدف بررسی همه‏گیری‏شناسی اختلالات روانی در افراد بالای 18 سال در مناطق شهری و روستایی استان لرستان در سال 1380 انجام گرفت. مواد و روشها: نمونه مورد مطالعه به روش تصادفی خوشه‏ای و منظم از بین خانوارهای موجود استان لرستان انتخاب گردید و با تکمیل پرسش‌نامه اختلالات عاطفی و اسکیزوفرنیا (SADS) توسط کارشناسان روان شناسی در استان، جمعاً 546 نفر مطالعه شدند و براساس معیارهای طبقه‏بندی DSM-IV، تشخیص‏گذاری اختلالات صورت گرفت. یافته ها: نتایج این بررسی نشان داد شیوع انواع اختلالات روان پزشکی در استان 05/19 درصد بوده که این شیوع در زنان 81/23 درصد و در مردان 29/14 درصد بوده است. اختلالات اضطرابی و خلقی به ترتیب با 46/12 و 48/3 درصد، شایع‏ترین اختلالات روانپزشکی در استان بود. شیوع اختلالات روان پریشی در این مطالعه 48/1 درصد، اختلالات عصبی- شناختی 47/1 درصد و اختلالات تجزیه‏ای 18/0 درصد بوده است. در گروه اختلالات خلقی، افسردگی اساسی با 2/2 درصد و در گروه اختلالات اضطرابی، اختلال اضطراب منتشر با 68/5 درصد شیوع بیشتری داشته ‏است. نتیجه گیری: در این مطالعه 36/11 درصد افراد تحت بررسی دچار حداقل یک اختلال روان پزشکی بوده‏اند. شیوع اختلالات روان پزشکی در استان در افراد گروه سنی 60-24 سال با 57/14 درصد، افراد طلاق گرفته یا جدا شده با 25 درصد، افراد ساکن در شهر خرم‌آباد با 85/14 درصد، افراد دارای تحصیلات عالی با 51/19 درصد و افراد بازنشسته با 75/18 درصد بیش از گروه‌های دیگر بوده است. لذا نتایج این تحقیق مسئولیت سیاست گذاران و برنامه‌ریزان بهداشتی استان لرستان و کشور را در رابطه با تدوین برنامه‌های عملی و اجرایی بهداشت روان بیش از پیش روشن می‌سازد.

Title: تأثیر توأم سیتوتوکسیک وروتوکسین تیپ 1 و منوفسفوریل لیپیدA بر روی رده سلولی M‏CF-7 در شرایط آزمایشگاهی

http://yafte.lums.ac.ir/article-1-992-fa.html

مقدمه: وروتوکسین ها سمومی با ساختمان دو بخشی هستند که در پاتوژنز سویه های اشرشیا کولی انتروهموراژیک دخالت دارند. مطالعات اولیه درمان برخی تومورهای سرطانی و نیز بررسی اثر سیتوتوکسیسیته این سموم روی رده های توموری در شرایط محیط کشت (In Vitro) وحیوانات آزمایشگاهی نشان دهنده اثر بخشی آنهاست.این سموم به دلیل دارا بودن گیرنده های اختصاصی بر روی رده های توموری مطالعه شده به صورت انتخابی عمل می نمایند. هدف از این تحقیق بررسی سیتوتوکسیسیته وروتوکسین1 تخلیص شده از سویه مولد وروتوکسین اشریشیا کولی توأم با منوفسفوریل لیپیدA (MPL) بر روی رده سلولی تومور پستانی انسان به نامMCF-7 بود. مواد و روشها: در این تحقیق ابتدا وروتوکسین1 تولید شده از 5 سویه مولد وروتوکسین به دست آمده از انستیتو پاستور ایران و آزمایشگاه مرجع بو علی با استفاده از کیت آگلوتیناسیون معکوس (VTEC-RPLA) تأیید و با استفاده از کروماتوگرافی جذبی تخلیص شد. در مرحله بعد رقت های مختلف وروتوکسین1 و MPL به صورت توأم و یا جداگانه بر روی رده سلولی MCF-7 اضافه شد و قابلیت زنده ماندن سلول ها Viability) ( با استفاده از تریپان بلو و شکستگی ژنوم آنها با الکتروفورز آگارز بررسی گردید. یافته ها: مطالعات ما نشان داد که سلول های MCF-7 مطابق با تحقیقات قبلی حساسیت فوق العاده ای به وروتوکسین1 دارند و مقدار 33 نانوگرم در هر میلی لیتر اثری معادل دوز سیتوتوکسیک 50 درصد CD50% ایجاد می کند. بررسی های شکستگی ژنوم سلولی الگوی مشخصی از بروز پدیده آپوپتوزیز را نشان می دهد. در حالی که MPL به تنهایی ماده ای غیر سمی است؛ ولی استفاده توام آن با وروتوکسین1 اثرات سمی آن را تشدید می کند. نتیجه گیری: اگرچه اثرات ضد توموری وروتوکسین1 از سال ها قبل روشن شده است؛ ولی کشف و شناسایی عوامل مختلف تشدید کننده اثر سیتوتوکسیک مواد ضد تومورها نیز مورد توجه محافل علمی است.MPL یکی از متابولیت های میکروبی است که اثر سینرژیستی آن با وروتوکسین1 در مرگ سلول های توموری پستان برای اولین بار در این تحقیق مورد بررسی شده است.

Title: بررسی موارد گچ خوری به عنوان یک روش جدید و عجیب اقدام به خود کشی در استان لرستان

http://yafte.lums.ac.ir/article-1-993-fa.html

مقدمه: «خودکشی با گچ» یا« خوردن گچ» یک روش جدید و عجیب از انواع راه های اقدام به خودکشی است که در چند سال اخیر فقط در استان لرستان و حواشی آن مشاهده شده است. با توجه به ناشناخته بودن این پدیده، ویژگی های افراد خودکشی کننده با گچ، علائم و نشانه های بیمار، نحوه برخورد و روش های درمانی مناسب، مطالعه ای با هدف بررسی موارد خودکشی با گچ صورت گرفت. مواد و روشها: در این مطالعه توصیفی ـ مقطعی، بیماران گچ خورده مراجعه کننده به بیمارستان شهدای عشایر خرم آباد طی سال های 74 تا 79 بررسی شدند. ابزار گردآوری اطلاعات پرسشنامه ای حاوی سوالاتی راجع به داده های دموگرافیک افراد، علائم، نشانه ها، روش های درمانی انجام گرفته و نتایج اقدامات بود. در مجموع 40 بیمار مورد مطالعه قرار گرفت. از تعداد 40 مورد بررسی شده 5/57% زن و 5/42% مرد بودند. بیشترین موارد اقدام به خودکشی با گچ در گروه سنی 10 تا 19 سال قرار داشتند (45%). این روش در تابستان بیشتر شایع بوده است و اکثریت اقدام کنندگان را دانش آموزان تشکیل می دادند (5/37%). نیمی از گچ خورده ها تحت درمان شستشوی معده با نرمال سالین قرار گرفته بودند که از این تعداد فقط یک بیمار نیاز به جراحی پیدا کرده بود. در مجموع فقط 7 بیمار از کل بیماران تحت عمل جراحی (لاپاراتومی+ گاستروتومی+ خروج توده گچ+ گاسترورافی) قرار گرفتند. نیمی از بیماران فاقد هرگونه علامت و نشانه بودند؛ اما در بیماران علامت دار، بیشترین علامت درد شکم (5/42%) و بیشترین نشانه لمس توده در شکم (5/17%) بود. نتیجه گیری: به نظر می رسد شستشوی معده با محلول نرمال سالین با مقادیر بیش از 2 لیتر (بلافاصله در بدو ورود) و سپس درمان نگهدارنده و پیگیری بیماران با معاینات روزانه و عکس برداری ساده از شکم به طور متوالی در این بیماران راهکار درمانی مناسبی باشد و جراحی تنها در بیمارانی که دچار علائم انسداد معده و روده و یا توده شکمی مراجعه کرده باشند کاربرد دارد.

Title: تغییرات طیف امواج مغزی و ناتوانی یادگیری و حافظه ناشی از تماس با سطوح پایین سرب در طی دوران جنینی در رات

http://yafte.lums.ac.ir/article-1-994-fa.html

مقدمه: مسمومیت با سرب در کودکان با عدم توانایی تکامل عصبی همراه بوده و ممکن است به نارسایی حرکتی وهوشی منجر شود. هدف از انجام مطالعه حاضر ارزیابی اثرات دراز مدت تماس با سطوح پایین سرب در طی دوران جنینی بر روی یادگیری، حافظه و امواج مغزی (EEG) بود. مواد و روشها: در طی دوران بارداری به راتهای مادر، استات سرب با سه دوز 05/0، 1/0 و 2/0 درصد و به گروه کنترل آب دوبار تقطیر داده شد. از نوزادان نر هرچهار گروه، آزمون رفتاری با استفاده از شاتل باکس و به روش اجتنابی فعال یک طرفه انجام شد. در این روش تعداد شوکهای الکتریکی بعنوان معیاری از یادگیری و حافظه در نظر گرفته شد. آزمون یادگیری در سن 60 روزگی وآزمون حافظه در سن 90 روزگی صورت گرفت. پس از انجام تستهای رفتاری، الکترودهای مورد نیاز در ناحیه حسی- حرکتی قشر مغز کاشته شدند و پس ا ز طی دوره بهبودی امواج مغزی ثبت شدند. یافته ها: نتایج بدست آمده نشان داد که سرب تاثیری بر یادگیری نداشته و تنها دوز 1/0 درصد آن سبب افزایش تعداد شوک دریافتی (98/0± 185/5) در مقایسه با گروه کنترل (62/0± 667/1) شده است. به عبارتی این دوز استات سرب سبب کاهش حافظه شده است .(P< 0.05) همچنین به استثنای یک افزایش در موج دلتا هیچ تفاوت معنی داری در امواج چهارگانه مغزی بین گروههای تست و کنترل مشاهده نشد. نتیجه گیری: نتایج حاصل از این تحقیق نشان داد که سرب در دوزهای مورد استفاده تأثیر معنی داری بر روی الگوی امواج مغزی نداشته است و سرب فقط در دوز 1/0 درصد بر رفتار تاثیر داشته و سبب کاهش حافظه شده است.

Title: تعیین مقدار متوسط PTT در جامعه شهری خرم آباد در گروه سنی

http://yafte.lums.ac.ir/article-1-995-fa.html

مقدمه: اختلال های انعقادی جزء مهم ترین دسته از بیماری هایی است که انسان به آن مبتلا می شود و هر ساله باعث مرگ و میر قابل توجهی در افراد جامعه می گردد. خونریزی از یک طرف و افزایش انعقادپذیری از طرف دیگر همواره خطراتی است که بیماران مختلف را تهدید می کند. هدف از انجام این پژوهش تعیین مقدار متوسط PTT در جامعه شهری خرم آباد بود. مواد و روشها: در این مطالعه از بین افراد گروه سنی 60-20 سال تعداد 298 مورد که هیچ گونه بیماری در هنگام آزمایش و سابقه بیماری های خونریزی دهنده یا اختلالات انعقادی را نداشتند و در عین حال از هیچ گونه داروی ضد انعقادی نیز استفاده نمی کردند انتخاب و مقدار PTT این افراد اندازه گیری شد. کلیه آزمایش ها به روش Tilt-Tube Test در یک آزمایشگاه مرجع به طریق استاندارد انجام گرفت. در ضمن برای کاهش خطای اندازه گیری تمام نمونه ها را یک نفر آزمایش نمود. یافته ها: در این مطالعه 298 نفر (149 نفر مرد و 149 نفر زن) مورد بررسی شدند. در مردان تحت مطالعه , مقدار متوسط PTT معادل 44/13ثانیه و حدود اطمینان آن 91/34-97/33 برآورد شد. در زنان تحت مطالعه , مقدار متوسط PTT معادل 59/34 ثانیه وحدود اطمینان آن بین 12/35-06/34 تعیین شد. در کلیه نمونه ها (زن و مرد) مقدار متوسط PTT معادل 51/34 ثانیه و حدود اطمینان آن 86/34-16/34 بود. مقایسه متوسط PTT در خرم آباد با کتب مرجع تفاوت معنی داری را نشان می دهد (PV<0.005). نتیجه گیری: پیشنهاد می شود که مقدار متوسط PTT حاصل از این مطالعه در آزمایشگاه های شهر خرم آباد استفاده شود.

Title: تأثیرکمی وکیفی بیماری دیابت موش باردار بر جنین ها در مرحله قبل از لانه گزینی

http://yafte.lums.ac.ir/article-1-996-fa.html

مقدمه: نوزادان مادران دیابتی در مقایسه با نوزادان مادران سالم نقص های مادرزادی، سقط های جنینی و تأخیر رشد داخل رحمی را به طور معنی داری بالاتر نشان می دهند. بیشترتحقیقات در گذشته در مرحله ارگانوژنز صورت گرفته است. در تحقیق حاضر عوارض سوء ناشی از افزایش قند خون و یا اختلالات متابولیکی دیابت در مرحله قبل از لانه گزینی بر روی جنین ها بررسی شد. مواد و روشها: در این مطالعه موش های صحرایی ماده به دو گروه مساوی(60= n) آزمایش وکنترل تقسیم شدند. در گروه آزمایش داروی استروپتوزوسین به میزان gr/kg 30 به صورت داخل صفاقی به مدت سه روز متوالی تزریق و وضعیت دیابتی ایجاد شد. حیوانات هر دوگروه با موش های نر هم نژاد هم قفس شدند. در صورت مشاهده پلاک واژینال، صبح روز بعد به عنوان روز اول حاملگی درنظر گرفته شد. موش های باردار در روز دوم , سوم و چهارم بارداری به روش قطع نخاعی کشته شدند و با محیط کشت M2 فلاشینگ لوله و شاخ رحم انجام شد. جنین ها به قطراتµl 25 محیط M16 منتقل شدند و سرعت تقسیمات کلیواژی و تعداد جنین ها با استفاده از آزمون Anova و کیفیت بلاستومر های جنین با آزمونChi-squre بررسی شد. یافته ها: تعداد تقسیمات کلیواژی در گروه دیابتی نسبت به گروه کنترل به طور معنی دار ی(P<0.001) کاهش یافت. به طوری که در روز دوم فقط 63% جنین های دیابتی در مقایسه با 8/94% جنین های گروه کنترل به مرحله 2سلولی رسیدند. در روز سوم1/38% جنین های گروه دیابتی در مقایسه با 2/52% گروه کنترل به مرحله 4 سلولی و در روز چهارم 5/8% جنین های گروه دیابتی در مقایسه با 2/78% گروه کنترل به مرحله مورولایی رسیدند. تعداد جنین ها با کیفیت مطلوب در گروه دیابتی بطور معنی دار ی(P<0.001) کاهش یافت؛ به طوری که در صد فراوانی کیفیت مطلوب (A) در گروه آزمایش نسبت به گروه کنترل, در روز دوم (43/74% به 5/95%), سوم (5/47% به 1/94%) و چهارم (5/8% به 9/88%) کاهش داشته است. نتیجه گیری: به طور کلی دیابت موش های حامله سبب افزایش معنی داری در کیفیت نا مطلوب جنین ها به صورت افزایش تخریب سلولی و کاهش تعداد جنین ها در مقایسه با جنین های گروه کنترل بسته به منظور در روز دوم, سوم و چهارم در مراحل قبل از لانه گزینی شده است.

Title: تأثیر استنشاق فلز روی در معدن بر عملکرد بعضی از دستگا ه های بدن

http://yafte.lums.ac.ir/article-1-997-fa.html

مقدمه: عوارض ناشی از مسمومیت با فلز روی طی مطالعات متعددی بررسی شده است. از جمله این عوارض فیبروز ریه متعاقب استنشاق روی است. در مورد عوارض مزمن استنشاق این فلز در کارگران معادن ابهاماتی وجود دارد. انجام معاینات غربالگری دقیق و صحیح شاغلین معادن روی نیاز به شناخت قبلی از فراوانی نسبی عوارض مزمن ناشی از تماس استنشاقی با این فلز دارد. این مطالعه به منظور شناسایی این عوارض و ارائه آزمون های غربالگری مناسب برای این جمعیت انجام گردید. مواد و روشها: در این مطالعه توصیفی از 400 کارگر شاغل در معدن روی «باما» در مجاورت شهر اصفهان، معاینه کامل بالینی به عمل آمد و آزمایشات لازم از جمله سطح سرمی روی انجام شد. یافته ها: بر اساس نتایج به دست آمده میانگین اشتغال کارگران 2/8 سال بود. حدود 36% از شاغلین فشار خون دیاستولی بالاتر از 80mmHg داشتند. شکایت از تنگی نفس فعالیتی در 22% وجود داشت؛ ولی اختلال در نمای اسپیرومتری در 9% افراد مشاهده گردید. میانگین سطح سرم روی mg/dl99/7 بود و تنها 13% سطح سرمی معادل مسمومیت داشتند. فراوانی 13% مسمومیت با روی و 36 % فشار خون بالا نشان دهنده عوارض مسمومیت فلز روی در سیستم قلبی ـ عروقی است. نتیجه گیری: عوارض تنفسی ناشی از استنشاق روی بعد از 8 سال به صورت ضایعه تحدیدی ریه مشاهده می شود که مؤید آسیب پارانشیم ریه است. غربالگری اصلی در این افراد باید بر اندازه گیری فشار خون ماهیانه و اندازه گیری حجم های ریه و هم چنین اندازه گیری روی در ادرار یا گلبول قرمز به طور سالیانه متمرکز شود.

Title: بررسی شاخص سمیت فاضلاب در تصفیه خانه فاضلاب جنوب اصفهان با استفاده از دافنیا مگنا

http://yafte.lums.ac.ir/article-1-998-fa.html

مقدمه : امروزه آزمایش های زیست آزمون در اقدامات کنترل آلودگی آب جایگاه ویژه ای به دارد. لذا با تعیین سمیت پساب و بررسی اثرات آن بر منابع آبی با استفاده ازروش زیست آزمونی می توان میزان ونوع تصفیه لازم برای رسیدن به شرایط مطلوب را برای آبزیان تعیین کرد. در این تحقیق شاخص سمیت فاضلاب تصفیه خانه جنوب اصفهان با استفاده از دافنیا مگنا مطالعه قرار گردیده است. مواد و روشها: در این مطالعه، آزمایش ها با استفاده از نمونه های فاضلاب برداشته شده از چهار محل شامل فاضلاب خام ورودی، قبل و بعد از حوض ته نشینی اولیه و بعد ازحوض ته نشینی ثانویه انجام گردید. دافنیای اولیه برای کشت از محیط زندگی طبیعی آنها به تعداد لازم صید گردید؛ سپس یکی از دافنیاهای صید شده، به تنهایی کشت داده شد. پس از آن نوزادهای دافنیای اولیه برای کشت انبوه استفاده گردید. پس از تهیه رقتهای لازم از نمونه های فاضلاب با انجام آزمایش های لازم غلظت کشنده 48 ساعته، یکای سمیت و بازده واحدهای مختلف و کل تصفیه خانه تعیین گردید. یافته ها: نتایج نشان داد که LC50-48h برای فاضلاب خام ورودی30 درصد حجمی وبرابر ATU33/3 بود. به همین ترتیب LC50-48h برای فاضلاب بعداز تصفیه مقدماتی، اولیه و ثانویه 32، 85 و 52 درصدحجمی و براساس یکای سمیت حاد به ترتیب 125/3، 92/1و76/1بود. بازده تصفیه مقدماتی در کاهش سمیت 6% محاسبه گردید. به همین ترتیب بازده تصفیه اولیه، ثانویه وکل تصفیه خانه در کاهش سمیت 4/38%،8 % و50 % به دست آمد. نتیجه گیری: اطلاعات حاصل از این تحقیق در مجموع مشخص می سازد که زیست آزمونی برای ارزیابی سمیت فاضلاب و کنترل آلودگی آب لازم است. بنابراین می توان زیست آزمونی را به عنوان یک روش مناسب برای ارزیابی اثر و کارایی فرایندهای مختلف تصفیه و کنترل سمیت به عنوان اطلاعات پایه برای پایش پسابها به کار برد. با توجه به گسترش روز افزون سیستم های تصفیه فاضلاب در ایران، ضروری است که در کشور یک استراتژی مناسب برای پایش پساب در کشور ضروری است و که نتایج این تحقیق و تحقیقات مشابه می تواند راهگشا باشد.

Title: مقایسه ای پرخاشگری، اضطراب و رشد اجتماعی در فرزندان نوجوان جانبازان PTSD و نوجوانان غیر فرزند جانباز شهرستان اصفهان

http://yafte.lums.ac.ir/article-1-999-fa.html

مقدمه: فرزندان جانبازان اعصاب و روان به طور دائم در معرض ضربات روانی غیر مستقیم ناشی از جنگ قرار دارند و از طرف دیگر به دلیل همانند سازی با الگوی پدر در معرض بروز علائم رفتاری مشابه آنان هستند. به همین دلیل بررسی وضعیت روانی و رشد اجتماعی این نوجوانان برای شناخت بیشتر مشکلات آنان و برنامه ریزی دقیق تر و آگاهانه تر برای رفع این مشکلات ضروری به نظر می رسد. مواد و روشها: در این مطالعه توصیفی ـ مقایسه ای میزان پرخاشگری، اضطراب و رشد اجتماعی 141 نفر از فرزندان جانبازان در مقطع دبیرستانی با استفاده از 3 پرسش نامه خود ایفا سنجیده شد و با همسالان آنها در دبیرستان های اصفهان مقایسه گردید. دو گروه از نظر جنس، سال تحصیلی، معدل سال قبل و درآمد خانواده همسان (Match) شده بودند. یافته ها: یافته های این مطالعه نشان داد که پس از کنترل اثر متغیر مخدوش کننده سطح تحصیلات والدین، میزان اضطراب و پرخاشگری در فرزندان جانبازان به طور معنی داری بالاتر از گروه دیگر بوده (05/0p<)؛ ولی رشد اجتماعی آنان تفاوت معنا داری نداشت. نتیجه گیری: بالاتر بودن سطح اضطراب و پرخاشگری از یک سو و وضعیت نه چندان مطلوب اقتصادی – اجتماعی خانواده های جانبازان که فرزندان آنان را بیش از پیش مستعد این گونه اختلالات روانی و رفتاری می کند، از سوی دیگر، اهمیت پرداختن به برنامه های پالایش بهداشت و روان و اقدامات مداخله ای را در این قشر متذکر می شود.

Title: فراوانی هیپوگلیسمی در نوزادان بستری شده با تشخیص اولیه سپسیس در بخش نوزادان و NICU بیمارستان شهید مدنی خرم آباد

http://yafte.lums.ac.ir/article-1-1000-fa.html

مقدمه : سپسیس نوزادی عنوانی است برای توصیف یک سندرم بالینی که عبارتست از پاسخ سیستمیک نوزاد به عفونت موجود در خون با عفونت اندام های دیگر بدن یا بدون آن و ممکن است بر متابولیسم بدن نوزاد از جمله میزان قند خون تأثیر گذار باشد. هدف از این مطالعه بررسی فراوانی هیپوگلیسمی در نوزادان بستری شده با تشخیص اولیه سپسیس در بخش نوزادان و NICU بیمارستان کودکان شهرستان خرم آباد در مدت شش ماهه دوم سال 1381 بود. مواد و روشها: این مطالعه به روش توصیفی ـ مقطعی انجام شد و جامعه پژوهش شامل کلیه نوزادان بستری شده (43 نوزاد) در بخش های نوزادان و NICU بیمارستان شهید مدنی خرم آباد با تشخیص اولیه سپسیس در طول مدت پژوهش (نیمه دوم سال 1381) بود. ابزار سنجش پرسش نامه مشتمل بر ثبت اطلاعات در زمینه محل بستری (بخش نوزادان و NICU)، سن، جنس، وزن، نتیجه کشت خون، میزان قند خون موقع بستری و نیز علائم بالینی مربوط به سپسیس و هیپوگلیسمی شامل وضعیت واکنش نوزاد، اشتهای نوزاد به شیر مادر و علائم سمپاتومیمتیک و نروگلیکوپنیک بود. یافته ها: از 43 نوزاد پذیرفته شده با تشخیص اولیه سپسیس، 34 نوزاد (1/79%) دارای کشت خون منفی، 9 نوزاد (9/20%) دارای کشت خون مثبت، 7 نوزاد (3/16%) دارای قند خون پایین تر از حد طبیعی (هیپوگلیسمی از نظر یافته های آزمایشگاهی) در بدو بستری بودند. همه نوزادان هیپوگلیسمیک (100%) در گروه سنی 0-7 روزه قرار داشتند، شش نوزاد (7/85%) پسر و یک نوزاد (3/14%) دختر، شش نوزاد (7/85%) در گروه نوزادان با وزن کم (وزن کمتر از 2500 گرم)، پنج نوزاد (4/71%) دارای دو علامت کاهش میل به شیر خوردن و کاهش واکنش های دیگر نوزادی بود. بیشترین علامت نروگلیکوپنیک به شکل آپنه در5 نوزاد (4/71%) از بین نوزادان هیپوگلیسمیک بروز نمود. بین هیپوگلیسمی و موارد کشت خون مثبت ارتباط معنی داری به دست نیامد. نتیجه گیری: استنباط نهایی از این مطالعه آن است که هر چند از هیپوگلیسمی در کتب مرجع به عنوان یکی از عوارض بیماری سپسیس نام برده می شود، این عارضه فقط در 3/16% بیماران بستری با تشخیص اولیه سپسیس در بدو بستری به ثبت رسید؛ لذا پیشنهاد می شود که درمان هیپوگلیسمی و لاجرم شروع انفوزیون سرم قندی هیپرتونیک (غلظت دکستروز10% یا بیشتر) در این ارتباط محدود به موارد تأیید شده این عارضه پس از دریافت سریع جواب قند خون ارسال شده بدو بستری باشد.
